# A Prospective Viewpoint on Neurological Diseases and Their Biomarkers

**DOI:** 10.3390/molecules27113516

**Published:** 2022-05-30

**Authors:** Mehrukh Zehravi, Janisa Kabir, Rokeya Akter, Sumira Malik, Ghulam Md. Ashraf, Priti Tagde, Sarker Ramproshad, Banani Mondal, Md. Habibur Rahman, Aurel George Mohan, Simona Cavalu

**Affiliations:** 1 Department of Clinical Pharmacy Girls Section, Prince Sattam Bin Abdul Aziz University, Alkharj 11942, Saudi Arabia; 2 Key Laboratory of Modern Chinese Medicines, China Pharmaceutical University, Nanjing 210009, China; janisa_kabir@yahoo.com; 3 Department of Global Medical Science, Wonju College of Medicine, Yonsei University, Gangwon-do, Wonju 26426, Korea; rokeyahabib94@gmail.com; 4 Amity Institute of Biotechnology, Amity University Jharkhand, Ranchi, Jharkhand 834001, India; smalik@rnc.amity.edu; 5 Pre-Clinical Research Unit, King Fahd Medical Research Center, King Abdulaziz University, Jeddah 21589, Saudi Arabia; ashraf.gm@gmail.com; 6 Department of Medical Laboratory Sciences, Faculty of Applied Medical Sciences, King Abdulaziz University, Jeddah 21589, Saudi Arabia; 7 Amity Institute of Pharmacy, Amity University, Noida 201301, India; tagde_priti@rediffmail.com; 8 Department of Pharmacy, Ranada Prasad Shaha University, Narayanganj 1400, Bangladesh; ramproshad131135@gmail.com (S.R.); banani091110@gmail.com (B.M.); 9 Faculty of Medicine and Pharmacy, University of Oradea, P-ta 1 Decembrie 10, 410087 Oradea, Romania; mohanaurel@yahoo.com

**Keywords:** neurological disorder, neuroinflammation, proteinopathies, biomarkers

## Abstract

Neurodegenerative diseases (NDDs) are disorders that affect both the central and peripheral nervous systems. To name a few causes, NDDs can be caused by ischemia, oxidative and endoplasmic reticulum (ER) cell stress, inflammation, abnormal protein deposition in neural tissue, autoimmune-mediated neuron loss, and viral or prion infections. These conditions include Alzheimer’s disease (AD), Lewy body dementia (LBD), and Parkinson’s disease (PD). The formation of β-sheet-rich aggregates of intra- or extracellular proteins in the CNS hallmarks all neurodegenerative proteinopathies. In systemic lupus erythematosus (SLE), numerous organs, including the central nervous system (CNS), are affected. However, the inflammatory process is linked to several neurodegenerative pathways that are linked to depression because of NDDs. Pro-inflammatory signals activated by aging may increase vulnerability to neuropsychiatric disorders. Viruses may increase macrophages and CCR5+ T cells within the CNS during dementia formation and progression. Unlike medical symptoms, which are just signs of a patient’s health as expressed and perceived, biomarkers are reproducible and quantitative. Therefore, this current review will highlight and summarize the neurological disorders and their biomarkers.

## 1. Introduction

Neurodegenerative diseases (NDDs) are disorders that affect both the central and peripheral nervous systems. Infection may play a role in late-onset AD, among the other risk factors causing sporadic occurrences of AD. Infectious agents such as bacteria, viruses, fungus, and protozoa have been linked to the development of Alzheimer’s disease (AD) over the last three decades [1,2]. The human brain is the world’s most complicated biological organ. We are not invincible, though, and we are susceptible to a variety of medical conditions, some of which are linked to a brain malfunction [3]. Neurodegeneration develops immediately before clinical manifestation of AD, i.e., the development of cognitive impairment, as a result of cumulative tau and Aβ pathologies, followed by a cellular malfunction in the brain [4]. Nervous system damage, ischemia, oxidative and endoplasmic reticulum (ER) cellular stress, inflammation, abnormal protein deposition in neural tissue, autoimmune-mediated neuronal loss, and viral or prion infections are just a few of the causes of NDDs [5]. Additionally, neurodegeneration comprises many clinically and pathologically heterogeneous disorders, most characterized by the accumulation of misfolded proteins in the CNS as insoluble aggregates (or inclusions) and gradual neuronal death in the affected regions [6,7,8]. AD symptoms include memory loss and personality changes, Parkinson’s disease (PD) symptoms include impaired motor capacity and focus challenges, and amyotrophic lateral sclerosis (ALS) symptoms include weakness and cognitive decline [2,9,10]. Multiple sclerosis (MS) is a peripheral nerve condition that includes diabetic neuropathy, various metabolic neuropathies, endocrine neuropathies, and disorders of myelin degeneration, all of which present with sensory impairments and autonomic dysfunction [11,12,13]. The research and development of effective therapy solutions for NDDs are the most complex and challenging topics in contemporary neuromedicine. Simultaneously, the social and economic benefits of resolving, at least in part, these issues outweigh any potential dangers or development costs. Although prevalent neurodegenerative disorders such as Alzheimer’s and Parkinson’s were described over a century ago, they remain incurable. Despite significant progress in understanding these diseases’ pathophysiology, the triggers and exact mechanisms of neurodegeneration remain unknown [14,15].

Biomarkers can be utilized for a variety of purposes, including guiding clinical diagnosis, estimating disease risk or prognosis, determining disease stage, and tracking medication response [16]. Biomarkers can be used in clinical trials to select a specific diagnostic subgroup (patient enrichment or stratification), assure proper treatment target engagement, detect therapeutic downstream effects on the disease process, and to assess clinical efficacy and/or safety [17]. The creation of blood-based biomarkers has been a significant advancement in the study of neurology. Despite initial skepticism about peripheral markers due to the physical constraints imposed by the blood-brain barrier (BBB), recent technical improvements have enabled the measurement of analytes in various biofluids at extremely low concentrations [18].

Numerous mutations mediating family variants of major neurodegenerative disorders have been discovered in several genes over the last two decades, igniting a fresh wave of research into the pathology of neurodegeneration and the mechanisms underlying neuronal protection [19,20,21]. Based on toxicological and transgenic disease models, a number of possible neurodegenerative routes have been postulated. They have similar characteristics and mechanisms, such as an accumulation of intrinsically disordered proteins in the form of aggregates, mitochondrial dysfunction and oxidative stress, and neuroinflammation, which have been used to develop therapeutic strategies aimed at halting or slowing disease progression rather than symptomatic treatment [22,23,24,25]. One of the outcomes of bacterial and viral infections is a disrupted BBB, which leads to widespread cerebral dysfunction after the systemic inflammatory response, with or without direct CNS infection [26]. Higher levels of the systemic inflammatory marker TNF-α have been linked to increased cognitive impairment in Alzheimer’s patients [27]. Microglia are activated after CNS infection by viruses, bacteria, fungi, and parasites, according to recent findings from both preclinical and clinical research. Because of their potential to acquire various activation states or phenotypes, microglia, a marker of brain inflammation, have several features for neuroinflammation, including cytotoxicity, repair, regeneration, and immunosuppression [28]. Recent research shows that infection with Enterobacteriaceae family bacteria accelerates the onset of AD in a Drosophila model by boosting immunological hemocyte migration to the brain [29].

However, many NDDs have identical symptoms and characteristics, making diagnosis difficult. As a result, a lot of study has been undertaken to investigate the clinical aspects as well as the molecular mechanisms that cause these diseases, to find traits that can help with diagnosis. Therefore, this current review will highlight and summarize the neurological disorders and their biomarkers.

## 2. Neurodegeneration, Inflammation, and Tumorigenesis in the Central Nervous System

Although the pathogenic function and basic molecular processes underpinning neurodegeneration are complicated, involving genetic, environmental, and endogenous variables linked with aging, their pathogenic function and basic molecular mechanisms are unknown [30,31]. Now, NDDs are categorized based on their known genetic pathways and the primary chemicals found in their protein deposits. These disorders are called ‘protein misfolding’ diseases or proteinopathies because significant structural abnormalities cause them in proteins [32,33]. Numerous fundamental mechanisms underlying neurodegeneration may be initiated at various stages of the neurodegenerative cascade by inflammatory cells and mediators. 

Apoptosis: Apoptosis is a form of planned cell death controlled by caspases [34,35,36,37]. It is characterized by the production of membrane-encased apoptotic bodies that are rapidly phagocytozed by macrophages or neighboring cells. Although evidence of apoptotic pathways has been found in animal models of a variety of neurodegenerative illnesses, there is less evidence in human tissues. In Huntington’s disease (HD) models, activation of caspase-1, -3, -8, and -9 as well as cytochrome c release were found in human striatal brain tissue. Similarly, in amyotrophic lateral sclerosis (ALS) and HIV-associated neurodegeneration, caspase activation and neuronal death have been observed [38]. 

Necroptosis: Necroptosis is a type of programmed cell death defined by the loss of plasma membrane integrity and occurs in the absence of caspase activation. The receptor-interacting serine/threonine-protein kinase 1 (RIPK1) and mixed-lineage kinase domain-like are the two key effector proteins in necroptosis (MLKL). TNF-α, FasL-, and TRAIL are released by astrocytes and can cause necroptosis by activating RIPK1 and MLKL, as shown in ALS mice models [39]. Axonal disease caused by RIPK1 was detected in pathological specimens from ALS patients [40]. In MS pathology samples, necroptotic pathways were also detected [41]. 

Autophagy, also known as type II programmed cell death, is defined by the buildup of autophagic vacuoles during cell death, along with potentially harmful components such as proteins or damaged organelles. Excessive autophagy can result in cell death and self-destruction. Autophagosomes were found in AD, HD, and PD patients’ damaged neurons. Numerous other triggers, such as food deprivation, mitochondrial toxins, hypoxia, and oxidative stress, can cause autophagy [42,43]. 

Axonal damage or transection can result in retrograde degeneration of the proximal neuronal cell body, which is associated with a range of degenerative alterations within the cell body, including apoptosis and neuronal perikaryon chromatolysis. Because of the relationship between neuronal apoptosis and axonal damage, inflammation-induced axotomy may result in retrograde (secondary) apoptosis of neuronal cell bodies [44]. 

Astrogliopathy: Astrogliopathy is a broad word that refers to astrocyte dysfunction. The abnormal buildup of inappropriately phosphorylated tau protein in astrocytes seen in AD, frontal temporal lobe dementia (FTLD), and corticobasal degeneration is referred to as aging-related tau astrogliopathy (ARTAG) [45,46]. Optic neuritis and myelitis characterize neuromyelitis optica (NMO), which can mimic MS. Antibodies against aquaporin-4 (AQP4), which binds to astrocyte water channels, are linked to NMO. NMO is characterized pathologically by a significant loss of immunoreactivity for the astrocytic proteins AQP4 and glial fibrillary acidic protein (GFAP), perivascular deposition of immunoglobulins, and complement activation, even in lesions containing some myelin [47]. 

Inflammation begins when the body’s immune cells start inflammatory cascades to avoid tissue damage caused by injury or invading pathogens. If the inflammatory response is successful, it eliminates pathogens, initiates wound healing and angiogenesis, and eventually decreases. When the neuroinflammatory response is acute, it is required and even helpful for the neuronal environment, as it aids in pathogen elimination and brain restoration. When serious threats to the neural environment, such as protein aggregates (Lewy bodies, neurofibrillary tangles), build in the brain and sustain inflammation for an extended length of time, continuous gliosis and apoptosis can occur as a result of uncontrolled inflammatory cytokine production. Chronic inflammation is associated to nearly all neurological diseases, including AD, PD, and ALS, as a result of persistent activation [48,49]. In contrast to this protective homeostatic mechanism, inflammation has been implicated in a wide variety of diseases. In recent years, its impact on neurological disorders has been hypothesized as a crucial role in disease progression. Microglia in the CNS form phagocytic morphologies and secrete pro-inflammatory cytokines to interact with astrocytes and neurons. This can result in neurodegeneration, synaptic phagocytosis, reduced neuronal function, microglial activation, inflammatory cytokine release, and even more microglial activation until the neural environment is no longer threatened. Astrocytes are also activated during the inflammatory process, a process known as astrogliosis. Aging is a significant risk factor for neurodegeneration [50,51,52]. In general, older adults have dysregulated cytokine expression (i.e., increased synthesis of pro-inflammatory cytokines and decreased availability of anti-inflammatory cytokines), resulting in a chronic low-grade inflammatory state. Inflammaging is a term that refers to this type of auto-inflammatory disorder that occurs throughout aging [53]. 

Aside from blood and lymphatic vessels, data suggests that neurogenesis (the growth of new neurons) and axonogenesis (tumor-induced neural sprouting toward the tumor microenvironment) are important in carcinogenesis and cancer progression. Neurogenesis and axonogenesis have been seen in pre-neoplastic lesions, implying that they play a role in the onset of cancer as an early occurrence in the pre-malignant phase [54].

## 3. Neurodegenerative Diseases as Proteinopathies

AD, LBD, and PD are the most frequent neurodegenerative proteinopathies. The formation of β-sheet-rich aggregates of intracellular or extracellular proteins in the CNS characterizes all neurodegenerative proteinopathies [55,56]. It is widely accepted that specific unstructured proteins in healthy brains change their shape in neurodegenerative proteinopathies, naturally undergoing severe structural folding and forming small oligomeric or large fibrillary clumps. These changes in size and three-dimensional shape cause self-association, elongation, and precipitation in certain brain regions, resulting in pathogenic protein properties being acquired. In most proteinopathies, the basic pathways generating misfolded protein structural alterations are similar. They may include post-translational alterations, decreased protein clearance, or increased protein production [57]. It has been claimed that protein clearance plays a vital function in maintaining neuronal cell integrity. Numerous studies have described how, in most neurodegenerative disorders, impaired protein clearance may affect brain functioning and structure, resulting in clinical symptoms [58,59]. Although adult neurons are thought to be terminally differentiated, degenerating neurons have been shown to accumulate linked cell-cycle-related proteins [60,61]. 

Because of the pathogenic mechanisms involving the transmission of the prion (the disease’s causative agent) or proteinaceous infectious particles, prion disease has a distinct phenotype among the proteinopathies. These particles are made up of a prion protein isoform (PrPSc) that is aberrant. The PrPSc isoform binds to PrPC fragments and causes conformational misfolding, resulting in autocatalytic amplification and transmission in the central nervous system [56]. As a result, prion illnesses are spread via highly infectious misfolded prions. The prion-like mechanism is based on irreversible connections between constitutive molecules and protein clearance resistance and the ability to propagate to target cells [57]. 

## 4. Relationship between Neurodegeneration and Inflammation

**Figure 1:**
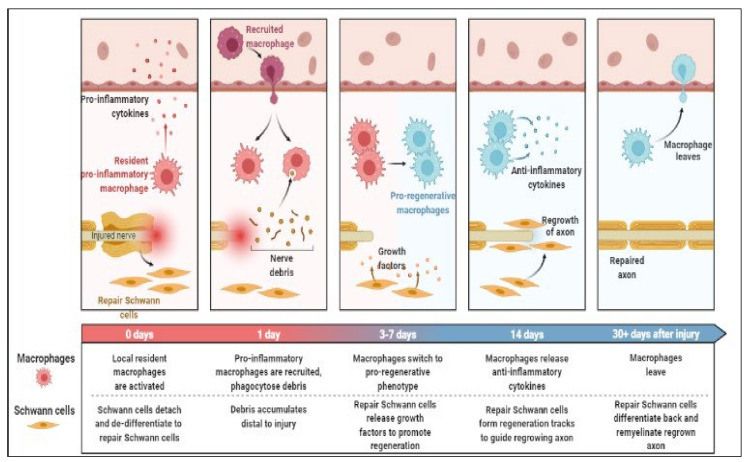
Inflammation and regeneration of peripheral nerves.

In mammals, the nervous system can directly sense inflammatory stimuli, allowing for the identification of a potential source of injury via the creation of pain and the modulation of the immune response to infection [62,63]. Although afferent routes and immunological information integration in the brain are still under investigation, there is evidence that central muscarinic signaling affects inflammation in experimental sepsis, obesity, and inflammatory colitis [64,65,66]. Inflammation and regeneration of peripheral nerves are shown in Figure 1.

The immune system is constantly on the lookout for possible infections and self-produced chemicals indicative of injury. Inflammation, under standard settings, is a well-coordinated reaction that is continuously fine-tuned. Once germs have penetrated the epidermal and mucosal barriers, innate immunity is crucial for limiting further invasion through the induction of inflammation. After the source of infection has been eliminated, the inflammatory response is critical for tissue repair and functional recovery. The precise systems that initiate and regulate inflammation will lessen the response if the source of the injury is removed. Large pathogen loads or infections with extremely virulent bacteria can cause sepsis and multiple organ failure [67]. 

SLE is an autoimmune disease that affects a range of organs, including the central nervous system, and is relapsing-remitting. It is characterized immunologically by a lack of tolerance for self-antigens and aberrant B- and T-cell responses. Immunoglobulin complexes can accumulate in tissues and cause systemic inflammation. Anti-nuclear antibodies are found in up to 98% of patients, but they can also be found in people with other autoimmune diseases. Neuropsychiatric SLE (NPSLE) is a poorly known medical condition with various clinical manifestations. According to one study, patients with neurological and mental disorders range between 12% and 95%. Involvement of the CNS predicts a more severe clinical manifestation of SLE [68]. Neuropsychiatric symptoms are challenging to diagnose due to their breadth of motor, sensory, cognitive, and behavioral manifestations [69]. 

The death of neurons in the hippocampus is linked to memory loss. Antibody-mediated cytotoxicity in response to binding to neuronal cell surface receptors such as the NMDA receptor or the neuronal surface P antigen (NSPA) may be the reason [70,71]. MS is the most prevalent demyelinating inflammatory illness in young adults, with a substantial risk of long-term disability. It affects approximately 2.5 million people worldwide [72]. 

Biological systems, such as the human brain’s neural network, exhibit “small-world” features. Small-world networks are organized on two layers. Locally, clusters of neurons specialized in a particular task create functional modules with a high degree of fast intramodular connection. On a global scale, many modules are connected via lengthy intermodular connections. The latter sort of connection benefits increased computational efficiency due to parallel data processing. White matter axonal fiber bundles generate long intermodular connections anatomically [73,74]. Long fibers have significant energy “wiring costs” [75]. The brain relies on a steady energy supply to maintain these long fibers. Recent research has revealed oligodendrocyte-derived lactate as the primary metabolic source for axonal maintenance undertaken in an elegant manner [76]. Disruption of this oligodendrocyte-neuronal metabolic coupling consistently results in neurodegeneration. Systemic inflammation significantly impairs the brain’s energy supply [77]. 

The human brain’s repair capacity is restricted, making it extremely sensitive to tissue injury. The brain is protected from many modes of tissue injury as central preventive mechanisms; for example, the skull bone protects the brain from mechanical harm, and the BBB protects the brain from blood-borne infections. Endothelial cells and astrocytes make up the majority of the BBB. Endothelial cells establish tight junctions, which provide a highly selective barrier that becomes permeable during systemic inflammation [78]. 

The buildup of misfolded Aβ in the brain has been suggested as the crucial initiating event in a complicated pathophysiological cascade leading to AD pathology. Robert Moir and Rudolph Tanzi demonstrated Aβ’s additional physiological role as an antibacterial agent in in vitro and in vivo experiments [79]. One study reported that systemic infection with the gram-negative bacterium *C. pneumoniae* was linked to a five-fold increase in the prevalence of AD, and higher anti-*C. pneumoniae* titers in the blood were also seen in many AD patients [80]. Therefore, after infection, the mice and *C. elegans* that expressed the Aβ peptide lived longer than those that did not. S. typhimurium injection in the brain resulted in the development of Aβ amyloid deposits with a longer survival rate in another A-overexpressing animal model. These findings also suggested that Aβ oligomerization, which is thought to be a pathogenic development in the context of neurodegeneration, could be a required step in increasing the peptide’s antimicrobial action [81]. Microbes provide an efficient surface for the nucleation of amyloid aggregates, increasing the likelihood of amyloid deposition [82]. Furthermore, Aβ buildup in the brain could be an early harmful event in the etiology of Alzheimer’s disease. The soluble and probably nontoxic Aβ monomers would form numerous complex assemblies with varying degrees of toxicity, including soluble oligomers and protofibrils. This could spread throughout the brain, eventually forming insoluble amyloid fibrils, which then combine into amyloid plaques, one of the hallmark histological lesions of AD. The biological importance of Aβ conformational states in the context of AD is crucial because different types of assemblies may influence the progression of neurodegenerative phases differently [83]. 

The immune system itself is a third possible source of brain tissue injury. Because pathogen defense is generally accompanied by host tissue damage, an anti-inflammatory environment protects the brain from abnormal immune activation. Astrocytes and neurons actively regulate the activation of brain immune cells in physiological settings [84]. 

## 5. Alzheimer’s and Neuroinflammation

The physiological and molecular pathways underlying neuroinflammation are most likely the same in aging and metabolic illnesses including hypertension, diabetes, depression, and dementia, as well as after brain injuries such as stroke, and are thus considered silent contributors to neuroinflammation [85]. Inflammatory pathways have been linked to the etiology of dementia and functional impairment in the elderly. Cerebral small vessel disease (SVD)-vascular dementia is hypothesized to be caused by systemic and local CNS inflammation, which results in persistent oligodendrocyte death and subsequent degradation of myelinated axons [86,87]. Another important risk factor for stroke and CNS tissue destruction is atherosclerosis, which is characterized by vascular inflammation caused by monocyte infiltration into the injured vascular wall and an increase in interleukin (IL)-6 levels associated with subsequent progression of intracranial significant artery stenosis following a stroke episode [88]. Inflammatory indicators including C-reactive protein (CRP) have also been detected in SVD. CRP is a potent predictor of subclinical and clinical atherosclerosis, as well as the progression of hemorrhagic stroke, in cardiovascular disease [89,90]. 

Furthermore, fatty tissue dysfunction associated with obesity and hypertension leads to low-grade inflammation, predisposes people to type 2 diabetes and cardiovascular disease, and may be linked to poor outcomes in stroke patients [91,92,93]. In diabetes mellitus, mortality is primarily due to micro- and macrovascular problems and sensory neuropathic disorders, which exacerbate the consequences of vascular disease. Sensory neuropathy contributes to the development of foot ulcers and eliminates warning signals associated with a heart attack. However, inflammatory, metabolic illness (metaflammation) related to poor nutritional habits can result in various ailments and diseases, including cardiovascular disease, stroke, hypertension, insulin resistance, metabolic syndrome, and diabetes mellitus. Metaflammation is characterized by the development of negative regulatory responses in target cells such as macrophages, lipid hormones (sphingolipids and eicosanoids), cytokines, and adipokines [94]. 

## 6. Depression and Neuroinflammation

Chronic activation of pro-inflammatory signals throughout aging may enhance sensitivity to neuropsychiatric diseases [95]. Inflammation was associated with more pro-inflammatory markers such as IL-6, CRP, and adipokines in obese women [96]. These pro-inflammatory indicators were related to depressive and anxious symptoms [97]. According to those findings, metabolic illnesses such as obesity, hypertension, and advanced age all serve as significant risk factors for depression, cognitive dysfunction, and dementia [98], and individuals suffering from severe depression have an increased risk of developing aging-related disorders affecting the cardiovascular, cerebrovascular, neuroendocrine, metabolic, and immunological systems [99,100,101]. Potential biomarkers with their features are shown in Table 1.

Hyperglutamatergia, oxidative stress, enhanced pro-inflammatory cytokines IL-6 and IL-8, and uncoupling of endothelial nitric oxide synthase have all been proposed as mechanisms linking inflammation and depression. As a result, patients with major depressive disorder (MDD) [102,103,104], a serious mental illness associated with higher levels of inflammatory markers in the periphery, depression, and suicide mortality, have been found to have indirect indications of neurovascular dysfunction. Inflammatory characteristics such as chemokines, adhesion molecules, cytokines, and acute phase proteins are all linked to neurodegenerative illnesses such as MDD [105,106].

## 7. Infections and Neuroinflammation

It has been suggested that viruses can reproduce in macrophages and CCR5+ T cells within the CNS, as with HIV proteins gp120 and Tat, which can cause neuronal apoptosis via CXCR4-PKC increase and neuronal dysfunction via miRNA disruption, respectively [107,108,109]. Most notably, as with HIV infection, additional viral insults are associated with increased cytokine secretion, increased cholesterol, increased lipopolysaccharide (LPS) concentrations, insulin resistance, testosterone insufficiency, and APOE4, all of which contribute to CNS inflammation. As a result of persistent stress, age-dependent augmentation of the provocative response appears to be the major trigger mechanism for tissue damage associated with numerous age-related diseases [110]. Local inflammation is frequently triggered by BBB endothelial cells that are equipped with the molecular machinery to detect bacterial and viral antigens. The antigenic recognition of a broad number of conserved molecular determinants known as pathogen-associated molecular patterns (PAMPs; Hanke and Kielian, 2011) by pattern recognition receptors (PRRs) is the first line of defense against microbial invasion [111,112]. Peripheral variables such as the gut microbiome can influence the condition of NDDs [113]. Changes in normal flora composition are linked to severe NDDs that disrupt brain development, plasticity, and create behavioral issues [114].

## 8. New Potential Biomarkers

In contrast to medical symptoms, which are simply indications of a patient’s health as expressed and perceived by the patient, biomarkers, or “biological markers“. In 1998, the Biomarkers Definitions Working Group of the National Institutes of Health defined biomarkers as “evidence of any biological, pathogenic, or pharmacogenomic response to any therapy modification” [115]. Biological markers are any substances, structures, or processes that may be measured inside or outside the body and can influence any changes in the body or the chance of disease prevalence of neurological diseases [116]. 

### 8.1. Alzheimer’s Disease

**Figure 2:**
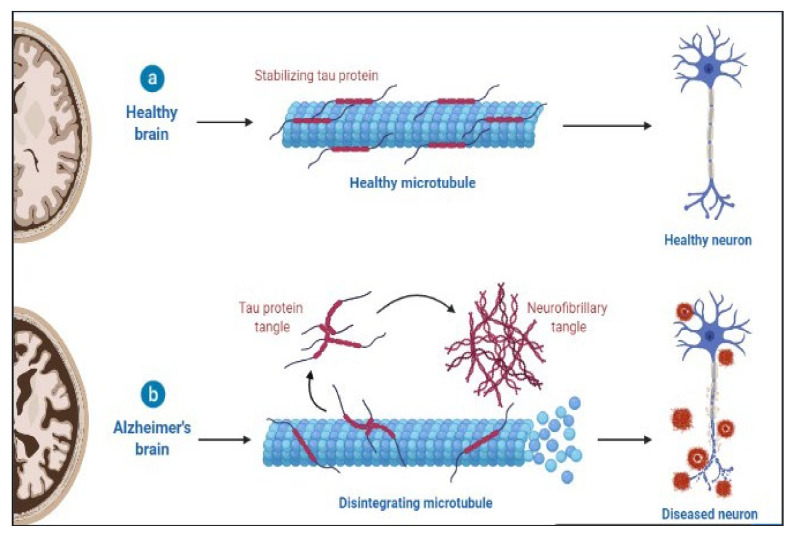
Pathology of AD.

AD is a slowly progressing neurological disease for which there is now no effective cure. Deposition of 42-amino-acid-long amyloid (Aβ) protein in extracellular plaques in the brain is the earliest identifiable disease, which occurs decades before clinical symptoms appear [117]. According to biomarker studies, a buildup is connected to synaptic dysfunction and increased tau phosphorylation and secretion, a microtubule-binding axonal protein abundantly produced in cortical neurons [118]. This dysregulated tau metabolism puts neurons at an elevated risk of degeneration, as intraneuronal neurofibrillary tangles formed of hyperphosphorylated and shortened tau proteins form. Neurodegeneration finally manifests as the AD clinical syndrome, characterized by progressive cognitive deficits [101,119,120]. The pathology of AD is shown in Figure 2.

### 8.2. Aβ Pathology Biomarkers 

Extracellular deposition of Aβ, formed by BACE1 and γ-secretase cleavage of amyloid precursor protein (APP), forming plaques is a significant pathological characteristic of AD. It has been hypothesized to represent the primary pathogenic event in the illness [124]. Aβ42 is an APP breakdown product generally transported from the brain interstitial fluid to the CSF and blood via the glymphatic system [125]. Amyloid positron emission tomography (PET) has been validated in comparison with neuropathology, has undergone substantial standardization in terms of quantifying pathology and defining abnormality cut-points, and has adequate usage criteria [126,127,128]. 

### 8.3. Tau Pathology Biomarkers 

A fundamental pathogenic hallmark of AD is the aggregation of hyperphosphorylated tau in the neuronal soma, generating neurofibrillary tangles. However, tau inclusions in neurons or glial cells are also observed in other neurodegenerative dementias [129]. Together with the CSF Aβ42/Aβ40 ratio, the cornerstone markers totaled tau (T-tau) and phosphorylated tau (P-tau) have been proposed as biomarkers for biologically defining AD and are considered diagnostic in the research criteria for AD [130,131]. Both T-tau and P-tau concentrations in the cerebrospinal fluid (CSF) reflect AD pathogenesis in all neurodegenerative dementias [132]. The most likely explanation is that the higher tau levels in the CSF result from enhanced tau phosphorylation and release by neurons in response to Aβ exposure [133,134]. 

### 8.4. Multiple Sclerosis

**Figure 3:**
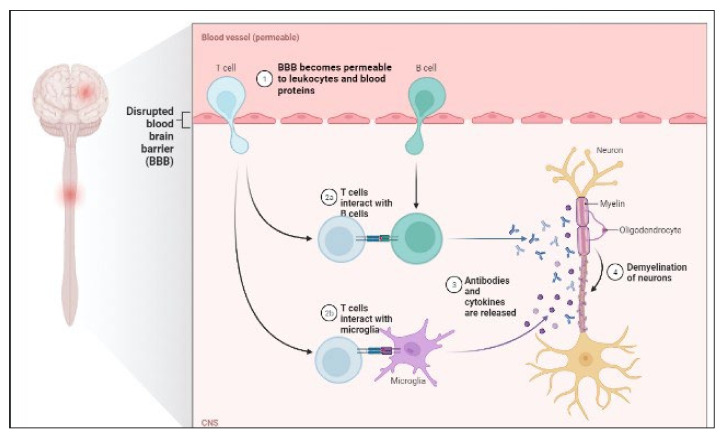
Pathology of MS.

MS is a chronic autoimmune disease that causes demyelination of the central nervous system and neurodegeneration [135]. Through interplay with immune cells, energy metabolic problems and endocrine abnormalities have been demonstrated to begin MS [136]. Furthermore, viral infection and environmental pollutants have been demonstrated to impair immunological tolerance and trigger the release of proinflammatory factors such as IL-6 and NF-kB in hereditarily vulnerable people [137]. The disease is heterogeneous in radiological and histological alterations, clinical presentation and development, and response to therapy [138,139]. P-gp expression is also increased, which promotes CD4+ and CD8+ T cell migration and amplifies neuroinflammation [140]. T-cells and B-cells mediate inflammatory responses by secreting cytokines that activate inflammatory cells such as microglia behind the BBB [141]. The pathology of MS is shown in Figure 3.

When a patient’s blood serum and CSF fluid are analyzed at the same time, oligoclonal bands are discovered. It has long been known that oligoclonal bands (OCB) can be found in the CSF of MS patients (by isoelectric focusing). Plasma cells in the CNS use immunoglobulin G (IgG) and M to create them (IgM) [142]. 

After a period in which OCB were not employed for diagnosis according to the McDonald criteria, they have been reintroduced into the diagnostic algorithm in the 2017 update. This shift toward substituting a positive CSF result for dissemination in time rather than in space is pragmatic. However, it underlines clinical neurologists’ responsibility to obtain cutting-edge CSF tests. MS is the most likely diagnosis for patients with typical clinical presentations, typical lesions, and alternative diagnoses that have been ruled out. By demonstrating the presence of OCB, we may provide proof for the disease’s immunological and inflammatory nature without waiting for the spread to occur. Thus, OCB is a well-established biomarker with clinical relevance for MS diagnosis [143,144,145,146]. 

#### 8.4.1. IgG Index

The immunoglobulin (Ig) G index is defined as the ratio of IgG’s CSF/serum quotient to albumin’s CSF/serum quotient. The albumin quotient, defined as albumin in CSF divided by albumin in serum, is used to assess blood-CSF barrier failure in MS [147]. The IgG index is used to quantify intrathecal immunoglobulin synthesis. An IgG index result greater than 0.7 implies an elevated intrathecal B cell response and, consequently, MS’s existence. Around 70% of people with MS have a high IgG index. As a result, this biomarker’s sensitivity is lower than that of the OCB [148,149]. 

#### 8.4.2. Antinuclear antibodies

Antinuclear antibodies (ANA) are tissue-independent autoantibodies directed against components of the cell nucleus that are quantified in the serum [150]. A continuously high titer indicates collagenous SLE [151]. 

#### 8.4.3. Anti-MOG antibodies

MOG is a myelin protein that is mostly located on the surface of myelin sheaths and oligodendrocyte membranes, and it could be a target for the autoimmune response in demyelinating disorders [152,153]. Therefore, anti-MOG antibodies, contrary to common perception, are only beneficial for differential diagnosis, not for MS diagnosis or prognosis. Using cutting-edge detection technologies, anti-MOG antibodies were found in a subset of pediatric patients with acute disseminated encephalomyelitis (ADEM), patients with clinical symptoms of NMOSD, and patients with bilateral optic neuritis in particular (cell-based approaches) [154]. 

#### 8.4.4. Anti-aquaporin-4 antibodies

Aquaporin-4 (AQP-4) is a water channel protein that is expressed by astrocytes in the CNS and is necessary for brain water homeostasis [155,156]. Antibodies to this protein are found in around 75% of patients with neuromyelitis optica spectrum disorder (NMOSD), but not in MS patients. Anti-aquaporin-4 antibodies are thus appropriate for high-specificity biomarkers. It is the first molecular biomarker to be clinically proven for differentiating between distinct CNS inflammatory demyelinating disorders. Antibodies against aquaporin-4 are frequently seen in the serum of patients suspected of having NMOSD [157].

## 9. Conclusions

Neurodegeneration is a condition that affects the CNS and is characterized by the loss of neuronal structure and function. One of the greatest challenges of our time is to disrupt neurological diseases, which pushes our clinicians and scientists every day. The presence of biomarkers aids in the early treatment of particular diseases. However, there is much research required to find a more advanced solution. Overall, this present review provides a unique opportunity to advance knowledge and innovation in the field of neurological biomarkers.

## Figures and Tables

**Table 1:** Potential biomarkers with their features.

Amyloid-Beta	Tau Protein	Phosphorylated Tau
Aβ plaque depositions commonly define AD. The amyloidogenic pathways produce these 42-amino-acid peptides (Aβ_1_-42), clumping in the brain. The amount of Aβ in AD patients’ CSF is reduced by roughly 500 pg/mL compared with healthy controls (79,420 pg/mL) [121].	Tau inclusion intraneuronal microtubule-associated protein is another well-established AD biomarker. There is an exponential increase in tau protein levels in AD patients from 300 to 600 pg/mL, which grows with age from 21–50 years to >71 years (in patients aged 51–70 years). Therefore it is an excellent prognostic biomarker [122].	In AD, tau protein is phosphorylated in about 39 places. Position 181 is a distinct biomarker in AD versus controls. Tau protein phosphorylation causes function loss and neuronal malfunction. There are also phosphorylated tau-199, tau-231, tau-235, tau-396 and tau-400 [123].

## Data Availability

Not applicable.
